# DOES CAREGIVING HASTEN AGING? EXPLORING THE LINK BETWEEN TRIPLE-DUTY CAREGIVERS’ TRANSITIONS AND SUBJECTIVE AGE

**DOI:** 10.1093/geroni/igae098.3253

**Published:** 2024-12-31

**Authors:** Xin Sun, Zhan Hu

**Affiliations:** Fudan University, Shanghai, Shanghai, China (People’s Republic); Fudan Institute on Ageing, Shanghai, Shanghai, China (People’s Republic)

## Abstract

Perceptions of aging serve as crucial indicators for evaluating positive aging. This study examines the relationship between various caregiving transition types and subjective aging over time. Utilizing data from the 2018 and 2020 waves of the China Longitudinal Aging Study, we examine six transition types, incorporating both intra- and extra-familial caregiving behaviors, by constructing three subjective aging change patterns: “Consistent over time”, “Accelerated growth”, and “Relative decline”. The findings reveal that sustained caregiving is associated with a relative decline in subjective age, with a more pronounced effect observed in the reduction of ongoing extramural caregiving. Caregiver role transitions, such as exiting caregiving and transitioning between intra- and extra-familial caregiving, lead to an accelerated increase in subjective age, while entering caregiving aligns subjective age with the passage of time. In the context of caregiving role transitions, a positive self-perception of aging enhances the subjective age of female caregivers, whereas a positive general aging attitude reduces the subjective age of male caregivers. The study underscores the significance of caregiving experiences as crucial predictive factors for subjective aging. Establishing comprehensive family support policies throughout the caregiving lifecycle, promoting voluntary caregiving participation, can contribute to enhancing adaptive aging

